# Sexually-Transmitted/Founder HIV-1 Cannot Be Directly Predicted from Plasma or PBMC-Derived Viral Quasispecies in the Transmitting Partner

**DOI:** 10.1371/journal.pone.0069144

**Published:** 2013-07-09

**Authors:** Pierre Frange, Laurence Meyer, Matthieu Jung, Cecile Goujard, David Zucman, Sylvie Abel, Patrick Hochedez, Marine Gousset, Olivier Gascuel, Christine Rouzioux, Marie-Laure Chaix, for the ANRS PRIMO Cohort Study Group

**Affiliations:** 1 Equipe d'accueil 3620, Université Paris-Descartes, Sorbonne Paris Cité, Paris, France; 2 Unité d’Immunologie, Hématologie et Rhumatologie pédiatriques, Assistance Publique – Hôpitaux de Paris, Hôpital Necker-Enfants malades, Paris, France; 3 Institut national de la Santé et de la Recherche médicale, Centre de recherche en Epidémiologie et Santé des Populations U1018, Université Paris 11, Faculté de Médecine Paris-Sud, Service d’Epidémiologie et de Santé Publique, Assistance Publique – Hôpitaux de Paris, Hôpital Bicêtre, Le Kremlin-Bicêtre, France; 4 Institut de Biologie computationnelle, Laboratoire d'Informatique, de Robotique et de Microélectronique de Montpellier, Unité mixte de Recherche 5506, Centre national de la Recherche scientifique et Université de Montpellier 2, Montpellier, France; 5 Service de Médecine interne, Assistance Publique – Hôpitaux de Paris, Hôpital Bicêtre, Le Kremlin-Bicêtre, France; 6 Service de Médecine interne, Hôpital Foch, Suresnes, France; 7 Service des maladies infectieuses et tropicales, Centre Hospitalier Universitaire de Fort de France, Fort de France, Martinique, France; 8 Laboratoire de Virologie, Assistance Publique – Hôpitaux de Paris, Hôpital Necker-Enfants malades, Paris, France; INSERM, France

## Abstract

**Objective:**

Characterization of HIV-1 sequences in newly infected individuals is important for elucidating the mechanisms of viral sexual transmission. We report the identification of transmitted/founder viruses in eight pairs of HIV-1 sexually-infected patients enrolled at the time of primary infection (“recipients”) and their transmitting partners (“donors”).

**Methods:**

Using a single genome-amplification approach, we compared quasispecies in donors and recipients on the basis of 316 and 376 C2V5 *env* sequences amplified from plasma viral RNA and PBMC-associated DNA, respectively.

**Results:**

Both DNA and RNA sequences indicated very homogeneous viral populations in all recipients, suggesting transmission of a single variant, even in cases of recent sexually transmitted infections (STIs) in donors (n = 2) or recipients (n = 3). In all pairs, the transmitted/founder virus was derived from an infrequent variant population within the blood of the donor. The donor variant sequences most closely related to the recipient sequences were found in plasma samples in 3/8 cases and/or in PBMC samples in 6/8 cases. Although donors were exclusively (n = 4) or predominantly (n = 4) infected by CCR5-tropic (R5) strains, two recipients were infected with highly homogeneous CXCR4/dual-mixed-tropic (X4/DM) viral populations, identified in both DNA and RNA. The proportion of X4/DM quasispecies in donors was higher in cases of X4/DM than R5 HIV transmission (16.7–22.0% versus 0–2.6%), suggesting that X4/DM transmission may be associated with a threshold population of X4/DM circulating quasispecies in donors.

**Conclusions:**

These suggest that a severe genetic bottleneck occurs during subtype B HIV-1 heterosexual and homosexual transmission. Sexually-transmitted/founder virus cannot be directly predicted by analysis of the donor’s quasispecies in plasma and/or PBMC. Additional studies are required to fully understand the traits that confer the capacity to transmit and establish infection, and determine the role of concomitant STIs in mitigating the genetic bottleneck in mucosal HIV transmission.

## Introduction

The transmission of human immunodeficiency virus type 1 (HIV-1) and the establishment of a productive infection are complex biological processes, and the details of the mechanisms remain to be elucidated. Initial studies of sexually acquired HIV-1 infection suggested that viral populations in the acute phase are generally highly homogeneous genetically, in contrast to the more heterogeneous viral populations found in chronic infections [1]–[6]. These findings thus suggested that HIV-1 infection is associated with a transmission “bottleneck”. However, more recent studies have reported heterogeneous virus populations shortly after infection in African female sex workers [7]–[10] and in American men who have sex with men (MSM) [11]. These observation suggest that the routes and circumstances of infection may affect the complexity of the transmitted virus [12].

The differing findings concerning the complexity of viruses during the acute and early phases of HIV-1 infection probably result from a combination of factors, including differences in the experimental designs and the methodologies used. A common approach has been to identify subjects within the first months following infection and to derive HIV sequences by bulk or near-limiting-dilution PCR amplification of proviral DNA or plasma RNA followed by cloning, sequencing and phylogenetic analyses [1], [2], [7], [10], [12]. In 2008, Keele et al. devised a novel strategy for a more precise molecular identification and enumeration of transmitted HIV-1 genomes [13]. This method, SGA-direct amplicon sequencing, was recently applied to clinical cohorts of acutely infected individuals [13]–[17], and the findings indicated that approximately 80% of heterosexual subjects and 60% of MSM are productively infected by a single viral genome. Since most of these studies only characterized the transmitted viral population in recipients, little information was available about its relationship with virus circulating in the donor. Also, few of these studies compared the viral populations identified by analysis of both RNA and DNA samples from donor/recipient pairs.

We report a study of eight transmission pairs, each of them including a sexually-infected patient enrolled into the French ANRS PRIMO cohort at the time of primary HIV-1 infection (PHI) (“recipient”) and his/her HIV-1-infected sexually-transmitting partner (“donor”). SGA-direct amplicon sequencing in plasma RNA and PBMC-derived DNA samples was used to compare C2V5 *env* gene sequences of the quasispecies in the donors and recipients.

## Patients and Methods

### Ethics Statement

The Ethics Committee of Cochin Hospital approved the study, and all the patients gave their written informed consent.

### The ANRS PRIMO Cohort

The patients were defined as having PHI from a western blot (WB) profile compatible with ongoing seroconversion (incomplete WB with absence of antibodies to *pol* proteins) (94% of the patients), detectable plasma HIV RNA with a negative or weakly reactive ELISA (2%), or an interval of less than 6 months between a negative and a positive ELISA result (4%) [18]. The date of infection was estimated as the date of symptom onset minus 15 days or, in asymptomatic patients, the date of incomplete WB minus 1 month, or the midpoint between a negative and a positive ELISA result. Patients were enrolled if HIV infection was estimated to have occurred less than 3 months previously. All patients were antiretroviral (ART)-naïve when enrolled into the cohort. At enrolment, blood samples were collected for immunological and virological studies. Participants completed standardized questionnaires describing HIV-acquisition risk group and sexual behavior (including number and characteristics of sexual intercourses before diagnosis of PHI and history of sexually transmitted infections (STI)). Serological screening for syphilis (*Treponema pallidum* hemagglutination assay (TPHA) and Venereal Diseases Research Laboratory test (VDRL)), hepatitis B (HBV) and hepatitis C viruses (HCV) was performed at enrolment. In cases of positive HBV or HCV screening results, HBV DNA and HCV RNA were quantified using the COBAS®Ampliprep/COBAS®TaqMan®HBV v.2.0 assay (Roche, Meylan, France) and the Abbott RealTime HCV® assay (Abbott, Rungis, France), respectively.

This study involved patients enrolled during PHI in the PRIMO cohort, who were able to identify their partner likely to be the source of their HIV infection, and agreed to participate in the substudy. They were then asked to propose to their partner to have a questionnaire and a blood sample in the month following enrolment of the recipient. Samples from donors were collected and systematically screened for syphilis, HBV and HCV at the time of collection. Between March 1998 and October 2008, 17 donor/recipients pairs were enrolled in our cohort. We herein study 8 out of these pairs, from which plasma and PBMC samples from donors were available.

### Laboratory Methods

HIV-RNA was quantified with the Cobas Taqman HIV-1 v1.5 assay (Roche Diagnostics, Meylan, France) as recommended by the manufacturers (threshold of detection of 20 copies/ml). Cell associated HIV-1 DNA in whole blood samples was quantified using the real-time HIV-1 DNA assay (Biocentric, Bandol, France) with a detection limit of 5 copies/PCR.

Drug resistance was evaluated by amplifying and sequencing the HIV-1 *reverse transcriptase* (RT) and *protease* genes in plasma HIV-RNA samples obtained at enrolment [19]. Resistance to nucleoside RT inhibitors, non-nucleoside RT inhibitors and protease inhibitors was defined according to the 2012 ANRS HIV-1 genotypic resistance interpretation algorithm (www.hivfrenchresistance.org).

HIV-1 subtype was determined by phylogenetic analysis of the RT and V3 *env* sequences, based on sequence comparisons with previously reported representatives of group M including the reference sequences of subtypes and sub-subtypes, and all the CRF sequences available in the HIV database or genbank (up to CRF54_01B) (http://www.hiv.lanl.gov).

### Viral RNA Extraction and cDNA Synthesis

Viral RNA was extracted from each sample using a home-made protocol: the patient plasma sample (500 µl) was centrifuged at 16,000 rpm for 1 hour at +4°C. The supernatant was removed, and 600 µl of HCV LYS v2.0 (from the Amplicor® HCV Specimen Preparation Kit, Roche Diagnostics, Mannheim, Germany) was added, followed by an incubation during 10 minutes at room temperature. A 600-µl of volume of isopropanolol was added and RNA was precipitated at 14,300 rpm for 15 minutes at +20°C. The supernatant was removed and the RNA pellet was rinsed with 1,000 µl of 70% ethanol. The tube was centrifuged at 14,300 rpm for 5 minutes at +20°C. The supernatant was removed and the RNA pellet was resuspended in 30 µl of water. RNA was immediately subjected to first strand cDNA synthesis by the Titan One Tube RT-PCR System® and the Protector Rnase Inhibitor® (Roche Diagnostics, Mannheim, Germany) according to the manufacturer's instructions. Each first strand synthesis reaction included 0.4 µM of the antisense primer ED12 (see sequence below). In some experiments, a different antisense primer, Env8, was used (see sequence below). The reactions were incubated at 50°C for 30 minutes.

### Proviral DNA Extraction

The QIAamp DNA Mini Kit® (Qiagen SA, Courtaboeuf, France) was used according to the manufacturer's instructions to extract total DNA from PBMCs from samples collected at inclusion.

### Single Genome Amplification (SGA)

C2V5 *env* amplicons were obtained from plasma cDNA or genomic DNA using nested PCR amplification by SGA [20]. Input genomic DNA or plasma cDNA was diluted to a limiting concentration such that approximately one-third or less of all second-round reactions produced a positive *env* amplicon. At this dilution, approximately 90% of the reactions will have originated from a single virus genome in the reaction [21]. Taq DNA Polymerase® (Roche Applied Science, Mannheim, Germany) was used according to the manufacturer's instructions for nested PCR amplifications. C2V5 amplification (627-bp fragment) was performed using the following primer combinations: first round: sense primer ED3 (5'-TTAGGCATCTCCTATGGCAGGAAGAAGCGG-3'), antisense primer ED12 (5'-AGTGCTTCCTGCTGCTCCCAAGAACCCAAG-3'); second round: sense primer ES7 (5'-GTGAATTCCTGTTAAATGGCAGTCTAGC-3'; nucleotide position relative to HXB2 genome start: 6994->7021), antisense primer ES8 (5'-GTGAATTCCACTTCTCCAATTGTCCCTCA-3′; nucleotide position relative to HXB2 genome start: 7676->7648). In cases of misamplification, different primers were used (334-bp fragment): first round: sense primer Env31 (5'-CAGTACAATGTACACATGG-3'), antisense primer Env8 (5'-ATGGGAGGGGCATACATTG-3'); second round: sense primer Env7 (5'-AATGGCAGTCTAGCAGAAG-3'; nucleotide position relative to HXB2 genome start: 7008->7026), antisense primer ED33 (5'-TTACAGTAGAAAAATTCCCCTC-3'; nucleotide position relative to HXB2 genome start: 7381->7360). PCR were performed in a reaction volume of 50 µl with cycling parameters as previously published [22]. The amplified products were purified using a QIAquick PCR Purification Kit® (Qiagen SA, Courtaboeuf, France).

### DNA Sequencing

Nucleotide sequences were determined by direct sequencing according to the manufacturer's instructions (Applied Biosystems, Foster City, CA, USA). Electrophoresis and data collection were performed on an ABI 3130 Genetic Analyser sequencer® (Applied Biosystems). Individual sequence fragments for each amplicon were assembled and edited using Sequence Navigator software [23]. All chromatograms were inspected for sites of mixed bases (double peaks), which would be evidence of priming from more than one template or the introduction of errors in early cycles of PCR. Any sequence with evidence of double peaks was excluded from the subsequent analyses.

Nucleotide sequences have been submitted to GenBank (accession numbers [KF142735-KF143426].

### Sequence Alignments

Clustal X was used for sequence alignments [24]. Phylogenetic interrelationships among viral sequences were estimated using Neighbor-Joining trees [25], and maximum likelihood methods with BioEdit and MEGA4 integrated molecular evolutionary genetic analysis software [26], [27]. The reliability of the tree topology was estimated from 1,000 bootstraps replicates.

### Hypermutated Samples

Enrichment for APOBEC3G/F mutations violates the assumption of a constant mutation rate across positions, as the editing performed by these enzymes are base- and context-sensitive. Enrichment for mutations with APOBEC3G/F signatures was assessed using *Hypermut* 2.0 (www.hiv.lanl.gov). Sequences that yielded a p-value of 0.05 or lower were considered significantly hypermutated and excluded from subsequent analyses.

### Nucleotide Sequence Diversity Analysis

Pairwise sequence similarities were calculated with DNADIST (http://cmgm.stanford.edu/phylip/dnadist.html) using the Kimura two-parameter algorithm [28]. Each set of sequences was then inspected using the *Highlighter* v2.2.1 tool (www.hiv.lanl.gov).

### Estimation of the Date of Infection of the Donors

The date of the donors' HIV-1 infection was unknown. Two methods were used to estimate the date. First, a serum sample was sent to the French National Reference Center for HIV and tested for recent infection by EIA-RI [29], [30]; this single indirect enzyme-linked immunosorbent assay quantifies antibodies for TM (gp41) and V3 peptides and has been validated as being able to discriminate recently infected individuals from those with long-lasting infection.

Second, we used a probabilistic modeling approach. For each data set, the *env* sequences were aligned using MAFFT (L-INS-i option) [31]. Sites with more than 75% gaps were removed, and we estimated the dates of infection of chronic patients as the date of the most recent common ancestor (MRCA) of his sequences, calculated using the Bayesian, MCMC-based program BEAST v1.7 [32]. We assumed a GTR+I+Γ4 substitution model and a strict molecular clock with a fixed substitution rate of 6×10^−3^ substitutions per site and per year (a standard value for *env*, see, for example, [33]). We used a Bayesian skyride tree prior as a coalescent demographic model with time-aware smoothing [34]. MCMC simulations were run for 2×10^8^ chain steps with sub-sampling every 2×10^5^. Convergence of the chains and results were inspected using Tracer v1.5. ESS values were larger than 200 for all parameters and all data sets, except for two parameters (*prior* and *posterior*) with the MRT data set. We also tested a lognormal relaxed molecular clock, but obtained poor results as nearly identical sequences were separated by large divergence times, most notably among recipient sequences. As a consequence, the infection date of the recipient was close to the infection date of the donor, thus contradicting clinical evidences.

### Viral Tropism Determination

The SVM_Geno2pheno_ algorithm (available at: http://coreceptor.bioinf.mpi-sb.mpg.de/cgi-bin/coreceptor.pl) with a 10% false positive rate was used to determine HIV-1 co-receptor usage by each virus.

## Results

### Study Subjects

SGA-direct sequencing was used to identify and enumerate transmitted/founder *env* sequences in eight patients with PHI, who reported sexual exposure as their primary HIV-1 risk behavior and who denied injection drug use (Table 1). These eight patients included three women and one man infected through heterosexual exposure and four MSM. All were infected with subtype B strains without resistance to the three main classes of antiretrovirals. One patient (recipient#2) was coinfected with HBV and HCV and three others (recipients#1, 3 and 7) reported histories of STI in the 6 months preceding HIV diagnosis. At the time of the study, four of the subjects were ELISA+/WB indeterminate, one was ELISA+/WB+/p31- and three were ELISA+/WB+/p31+.

**Table 1:** Characteritics of the 8 recipients.

Recipient	1	2	3	4	5	6	7	8
Date of sample collection	2/12/2002	3/25/1998	01/02/1999	6/26/2000	3/1/1999	4/18/2007	6/16/1999	10/24/2008
Estimated time since infection (days)	33	37	37	40	43	85	32	38
HIV-1 RNA (log_10_ copies/mL)	5.93	4.70	4.44	5.52	6.07	4.01	4.83	4.62
HIV-1 DNA (log_10_ copies/10e6 PBMC)	3.25	3.12	3.15	2.86	3.64	2.76	3.84	ND
CD4 cell count (cells/mm3)	520	734	368	681	381	635	558	523
Viral subtype	B	B	B	B	B	B	B	B
Viral resistance	0	0	0	0	0	0	0	0
Age (years)	38	28	36	21	28	39	29	19
Gender (male/female)	M	M	F	M	F	M	M	F
Risk group	Het	MSM	Het	MSM	Het	MSM	MSM	Het
Country of birth	France	Chile	France	France	Algeria	France	France	France
Living place	France	France	France(West Indies)	France (West Indies)	France	France	France	France (West Indies)
Number of regular sexual partners *	1	1	1	1	1	0	0	1
Number of casual sexual partners *	0	15	0	0	0	5	3	0
Serological syphilis testing	–	–	–	–	–	–	–	–
**HBV testing**								
HBsAg	–	–	–	–	–	–	–	–
HBsAb	–	+	–	–	–	+	–	–
HBcAb	–	+	–	–	–	–	–	–
HBV DNA (log_10_ IU/mL)	n.d.	Und.	n.d.	n.d.	n.d.	n.d.	n.d.	n.d.
**HCV testing**								
HCV Ab	–	+	–	–	–	–	–	–
HCV RNA (log_10_ IU/mL)	n.d.	1.7	n.d.	n.d.	n.d.	n.d.	n.d.	n.d.
**Prior sexually transmitted infections** §	Condyloma	No	Vaginal candidiasis	No	No	No	Condyloma	No

* In the 6 months preceding PHI diagnosis.

§ Urethritis, rectitis, genital herpes infection, vulvo-vaginal candidosis, condyloma and/or syphilis.

PBMC = peripheral blood mononuclear cells; Het = heterosexual; MSM = man having sex with men; HBV = hepatits B virus; HBsAg = HBV surface antigen; HBsAb = HBV surface antibodies; HBcAb = HBV core antibodies; HCV = hepatitis C virus; n.d. = not done; und = undetectable.

Blood samples from the donors were obtained concomitantly (donor#4), 14 (donors#1-3 and 6-7) or 30 days (donors#5 and 8) after the enrolment of their respective recently infected partner (Table 2). Envelope sequences in these samples were analyzed: the EIA-RI test suggested that the donors had been infected less than 6 months previously in two cases (donors#3 and 7), and that the other six patients had long-lasting infections. These findings were confirmed by our modeling approach: donors#3 and 7 were estimated to have been infected 0.46 and 0.20 years, respectively, prior to collection of the blood sample and the remaining donors were estimated to have been infected between 2.66 and 11.17 years previously (Table 2). Histories of STI were not available for donors. However, the microbiological screening for coinfections was positive in five patients; the pathogens identified were syphilis (n = 2, donors #4 and 6), HBV (n = 4, #2, 3, 4 and 5) and HCV (n = 1, #5). There was no evidence of transmission of these infections to their partners.

**Table 2:** Characteritics of the 8 donors.

Donor	1	2	3	4	5	6	7	8
Date of sample collection (compared with that of the recipient)	2 weeks later	2 weekslater	2 weekslater	at the sametime	1 monthlater	2 weekslater	2 weekslater	1 monthlater
Date of diagnosis of HIV infection (compared with that of the recipient)	12 years before	1 week before	<2 months before	1 week later	1 year before	11 years before	<6 months before	n.a.
Time of infection (estimated with a EIA-RI test) (months)	>6	>6	<6	>6	>6	>6	<6	>6
Time of infection (estimated using a mathematical model) (years) (mean, range)	3.85(2.95–4.86)	2.66(2.06–3.36)	0,46(0.32–0.62)	9.41(7.51–11.39)	11.17(8.66–14.03)	4.14(3.05–5.41)	0.20(0.09–0.31)	7.18(5.39–8.86)
HIV-1 RNA (log_10_ copies/mL)	5.89	4.75	5.22	4.20	4.40	5.80	5.10	4.60
HIV-1 DNA (log_10_ copies/10e6 PBMC)	3.53	3.64	3.03	3.73	2.81	3.90	3.33	3.91
CD4 cell count (cells/mm3)	216	503	n.a.	200–500	n.a.	200–500	800	n.a.
Viral subtype	B	B	B	B	B	B	B	B
Viral resistance	0	0	0	0	0	0	0	0
Age (years)	40	n.a.	n.a.	n.a.	n.a.	n.a.	n.a.	n.a.
Gender (male/female)	F	M	M	M	M	M	M	M
Risk group	Het	MSM	Het	MSM	Het	MSM	MSM	Het
Country of birth	n.a.	Egypt	France	n.a.	n.a.	France	France	France
Serological syphilis testing	–	–	–	+	–	+	–	–
**HBV testing**								
HBsAg	–	–	–	+	–	–	–	–
HBsAb	–	+	+	–	–	+	+	–
HBcAb	–	+	+	+	+	–	–	–
HBV DNA (log_10_ IU/mL)	n.d.	Und.	Und.	1.3	Und.	n.d.	n.d.	n.d.
**HCV testing**								
HCV Ab	–	–	–	–	+	–	–	–
HCV RNA (log_10_ IU/mL)	n.d.	n.d.	n.d.	n.d.	5.9	n.d.	n.d.	n.d.

¶ Results based on the declaration of the recipient.

EIA-RI test = enzyme-linked immunosorbent assay for recent infection [29], PBMC = peripheral blood mononuclear cells; Het = heterosexual; MSM = man having sex with men; HBV = hepatits B virus; HBsAg = HBV surface antigen; HBsAb = HBV surface antibodies; HBcAb = HBV core antibodies; HCV = hepatitis C virus; n.d. = not done; n.a. = data not available; und = undetectable.

### HIV-1 *env* Diversity Analysis

**Figure 1:**
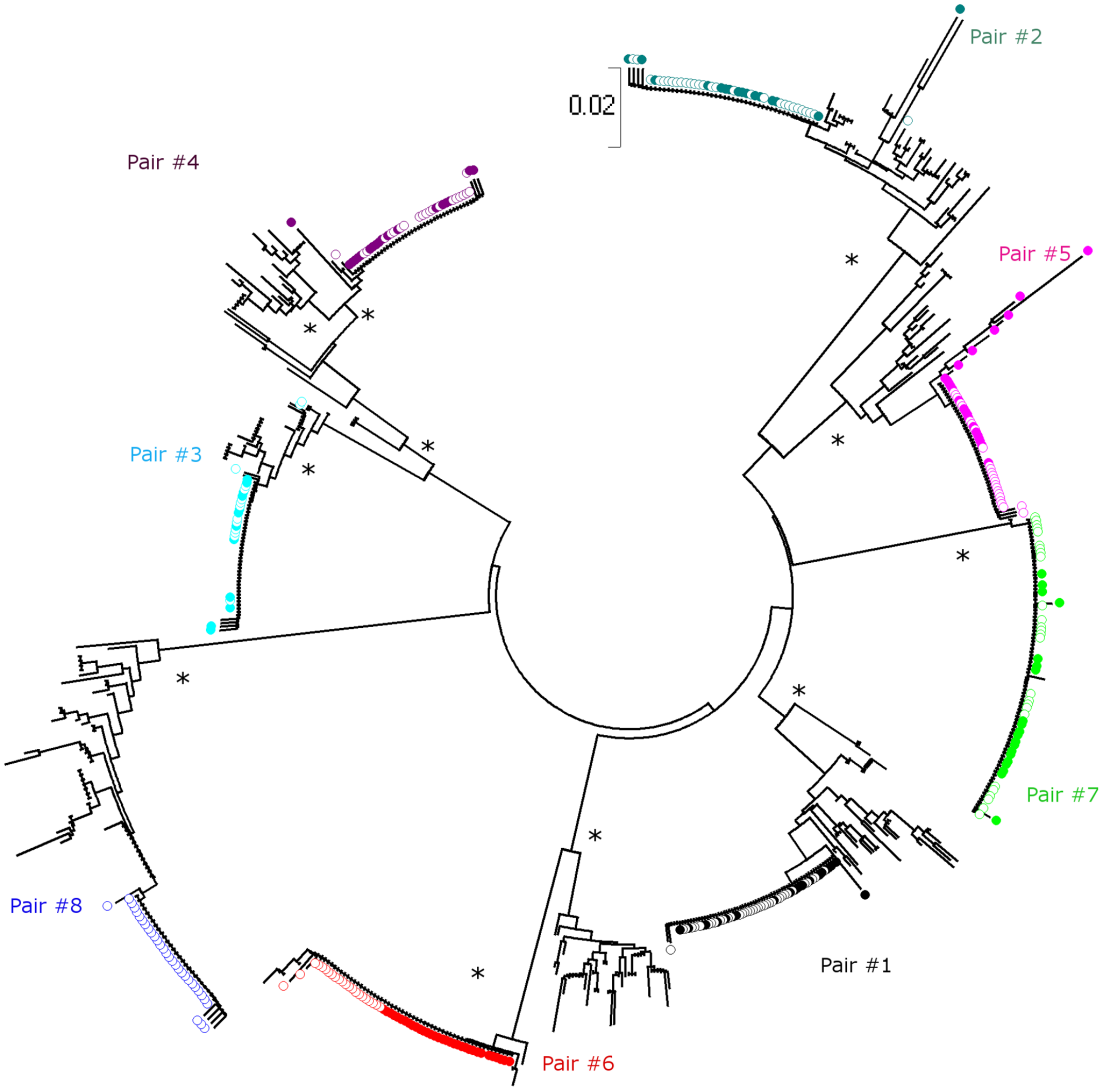
Evolutionary relationships between the HIV-1 *env* genes in the eight donor/recipient pairs. The evolutionary history was inferred using the Neighbor-Joining method [38]. The optimal tree with the sum of branch length = 2.01912678 is shown. The tree is drawn to scale, with branch length in the same units as those of the evolutionary distances used to infer the phylogenetic tree. The evolutionary distances were computed using the Maximum Composite Likelihood method [39] and the unit is the number of base substitutions per site. Codon positions included were 1^st^+2^nd^+3^rd^+noncoding. All positions containing gaps and missing data were eliminated from the dataset. There were a total of 230 positions in the final dataset. Phylogenetic analyses were conducted in MEGA4 [40]. For each recipient, viruses isolated from PBMC-derived DNA (•) and plasma RNA (○) are represented, with a different color for each donor/recipient pair. Asterisks indicate branches with bootstrap values greater than 98%.

**Figure 2:**
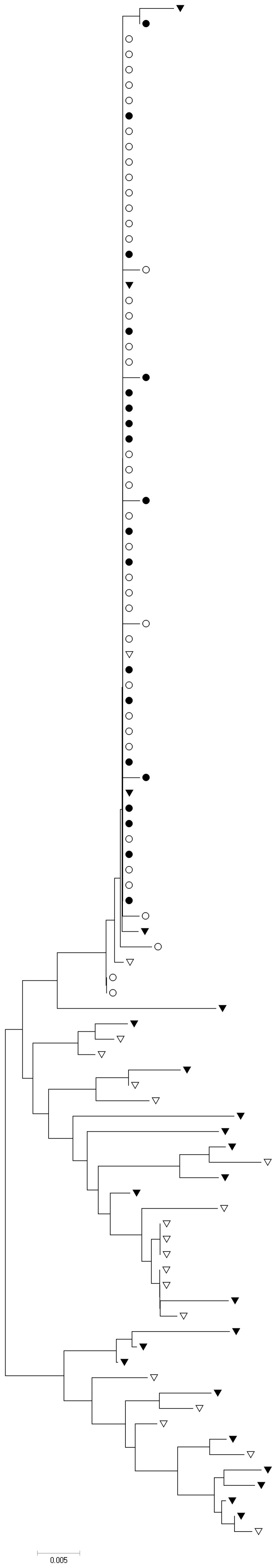
Evolutionary relationships between the HIV-1 *env* genes in donor/recipient pair #1. The evolutionary history was inferred using the Neighbor-Joining method [38]. The optimal tree with the sum of branch length = 0.28572580 is shown. The tree is drawn to scale, with branch lengths in the same units as those of the evolutionary distances used to infer the phylogenetic tree. The evolutionary distances were computed using the Maximum Composite Likelihood method [39] and the unit is the number of base substitutions per site. Codon positions included were 1st+2nd+3rd+Noncoding. All positions containing gaps and missing data were eliminated from the dataset (Complete deletion option). There were a total of 500 positions in the final dataset. Phylogenetic analyses were conducted in MEGA4 [40]. For the recipient, viruses from PBMC-derived DNA (•) and plasma RNA (○) are represented. For the donor, strains from PBMC-derived DNA (▾) and plasma RNA (∇) are represented.

Totals of 316 and 376 C2V5 *env* sequences were obtained from plasma vRNA and PBMC-associated DNA, respectively (median of 21 RNA sequences per subject; range 12–33; and 22.5 DNA sequences per subject; range 14–39). DNA *env* sequences could not be obtained from recipient#8 because appropriate samples were not available. A composite Neighbor-Joining phylogenetic tree was generated (Figure 1): the viral sequences formed eight distinct donor/recipient-pair-specific monophyletic lineages, each with strong statistical support (bootstrap values >98%), indicating that neither cross-contamination from other samples nor related transmission networks have occurred. Sequences from PBMC DNA and plasma RNA were distributed throughout the branch patterns of each donor and recipient, suggesting that these two sources were not compartmentalized. In all transmission pairs, recipient *env* sequences were highly homogeneous forming a distinct monophyletic subcluster within the tree of donor sequences. An example of the trees of donor/recipient pairs is given in Figure 2 (pair#1).

In 6/8 cases, the donor sequences were highly heterogeneous (mean within-patient *env* diversity = 4.03% (range 2.70–5.93)), consistent with the long-lasting infection of their host. By contrast, the *env* diversities were below 1% in donors infected less than 6 months ago (mean 0.51% and 0.14% in donors #3 and 7, respectively). Maximum within-patient *env* diversities for the eight recipients ranged from 0.04% to 0.34% for DNA sequences (mean 0.18%) and from 0.11% to 0.33% for RNA sequences (mean 0.17%) (Table 3). Mean within-patient *env* diversities were similar in cases of heterosexual and homosexual transmission (0.26% versus 0.14%, and 0.18% versus 0.13% for DNA and RNA sequences, respectively). All nucleotide diversity values were <0.75%, consistent either with single variant transmission or with transmission of only closely related viruses [13], [15]–[17], [35].

**Table 3:** Diversity and tropism analyses of V3 *env* sequences from 8 HIV-1 infected donor/recipients pairs.

Donor/recipient pair		Origin of sequences	No. Of SGA *env* sequences	APOBEC- mediated hypermutation	Nucleotide sequence diversity (%)		No. of CXCR4/DM-tropic virus	
					Mean	Range	n	%
1	Recipient	DNA	20a/21b	Yes	0.18	0.00–0.51	0/20	0.0%
		RNA	39/39	No	0.11	0.00–0.49	0/39	0.0%
	Donor	DNA	22/23	Yes	3.83	0.17–6.10	0/22	0.0%
		RNA	19/19	No	3.23	0.00–5.31	0/19	0.0%
2	Recipient	DNA	15/16	Yes	0.60	0.00–2.31	0/15	0.0%
		RNA	29/29	No	0.33	0.00–2.59	0/29	0.0%
	Donor	DNA	18/19	Yes	2.88	0.33–4.86	1/18	5.6%
		RNA	21/21	No	2.07	0.00–3.29	0/21	0.0%
3	Recipient	DNA	12/12	No	0.09	0.00–0.53	0/12	0.0%
		RNA	14/14	No	0.11	0.00–0.47	0/14	0.0%
	Donor	DNA	23/23	No	0.38	0.00–0.99	0/23	0.0%
		RNA	25/25	No	0.63	0.00–1.30	1/25	4.0%
4	Recipient	DNA	19/19	No	0.34	0.00–1.37	0/19	0.0%
		RNA	21/21	No	0.17	0.00–0.80	0/21	0.0%
	Donor	DNA	16/16	No	7.55	0.00–12.77	0/16	0.0%
		RNA	23/23	No	4.80	0.00–10.46	0/23	0.0%
5	Recipient	DNA	19/23	Yes	0.34	0.00–1.60	19/19	100.0%
		RNA	25/25	No	0.21	0.00–0.82	25/25	100.0%
	Donor	DNA	15/15	No	7.30	0.00–12.89	1/15	6.7%
		RNA	15/15	No	5.32	0.00–12.61	4/15	26.7%
6	Recipient	DNA	33/33	No	0.04	0.00–0.32	0/33	0.0%
		RNA	22/22	No	0.16	0.00–0.86	0/22	0.0%
	Donor	DNA	24/25	Yes	4.85	0.00–10.37	0/24	0.0%
		RNA	21/21	No	1.73	0.00–3.50	0/21	0.0%
7	Recipient	DNA	20/20	No	0.04	0.00–0.46	0/20	0.0%
		RNA	26/26	No	0.14	0.00–1.71	0/26	0.0%
	Donor	DNA	23/23	No	0.16	0.00–0.89	0/23	0.0%
		RNA	20/20	No	0.11	0.00–1.15	0/20	0.0%
8	Recipient	RNA	31/31	No	0.18	0.00–0.88	31/31	100.0%
	Donor	DNA	25/28	Yes	4.01	0.00–6.80	4/25	16.0%
		RNA	25/25	No	2.80	0.00–40.15	7/25	28.0%

a = initial sequence set.

b = total number of sequence analyzed after exclusion of APOBEC-mediated hypermutations.

Within-patient *env* diversities for the eight donors ranged from 0.16% to 7.55% (mean 3.92%) for DNA sequences, and from 0.11% to 5.32% (mean 2.44%) for RNA sequences. Again, viral diversity in both DNA (0.38%, 0.16%) and RNA sequences (0.63%, 0.11%) was low in donors#3 and 7, consistent with recent infection. The viral diversity in the six other donors was significantly higher in DNA than in RNA sequences.

**Figure 3:**
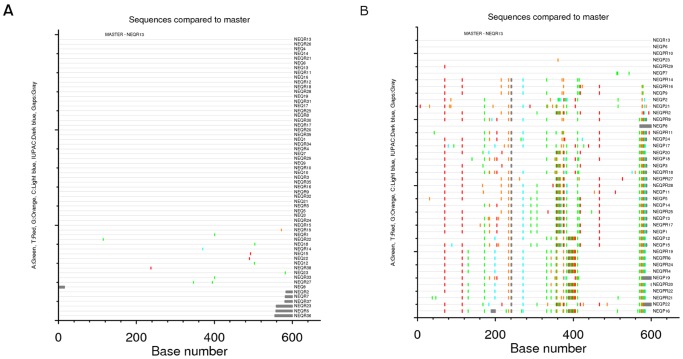
Comparative *Highlighter* analyses of *env* diversity in a donor-recipient HIV-1 transmission pair. (A) Recipient#1 shows evidence of infection with a single virus. (B) Donor#1 was the chronically infected partner of recipient#1. The same reference amplicon, a V3 RNA sequence from recipient plasma, was used to depict the viral diversity in both individuals.

To evaluate the extent of sequence diversity in recipients, *env* sequences from the recipients were examined using the *Highlighter* tool, which allows comparison of each recipient *env* sequence to a reference recipient sequence and graphically depicts all nucleotide differences between the two. An example of the output of this tool for one recipient (recipient#1) is given in Figure 3A; it shows a remarkable degree of homogeneity of the *env* sequences in this recipient, whose infection was estimated to have occurred 33 days prior to sample collection. Approximately 80% (47/59) of the sequences are identical despite each being amplified from a unique viral genome. Similar proportions of identical sequences were observed in sequences obtained from both plasma (32/39) and PBMC (15/20). Compared to the reference amplicon, approximately 17% of the sequences exhibited a single nucleotide change, and these changes were randomly dispersed over the C2V5 region; 2% of the sequences carried two nucleotide changes. Similar results were obtained by *Highlighter* analyses of the seven other recipients, consistent with single-variant transmission in all cases.

### Relationships between Recipient and Donor Quasispecies

The infection in each of the eight recipients appeared to have been established from a single donor variant. We sought to determine the frequency of this variant or closely related variants within the donor quasispecies and to examine whether such variants predominated in plasma- or PBMC-derived viruses in the donor. The number of nucleotide differences between each donor virus sequence and the consensus recipient virus sequence were calculated for each of the eight transmission pairs. In all cases, at least one donor variant was identified that differed by fewer than seven nucleotides from the consensus recipient variant (Table 4). The donor variants most closely related to the recipient sequences were found in both plasma (3/8) and PBMC (6/8) samples, such that it was not possible to establish whether the infecting virus was specifically derived from a single compartment. In the donor/recipient pairs #3 and 7, the viral quasispecies were largely homogeneous in both donors and recipients. In the other pairs, fewer than 20% of the donor sequences contained fewer than 10 nucleotide differences to the recipient consensus sequence. This is illustrated in Figure 3B, depicting the extent of sequence diversity in the donor#1 using the *Highlighter* tool, using the same reference amplicon as shown in Figure 3A (an *env* RNA sequence isolated from recipient plasma). These findings suggest than the virus establishing the new infection is derived from an infrequent circulating variant population in the donor.

**Table 4:** Relationship of donor variants to recipient consensus sequence.

Recipient	Number of donor variants analyzed	Blood compartment of originof nearest donor variant	Number of nucleotide differencescompared to nearest donor variant		Number of donor sequences<10 nucleotides different	
			in plasmacompartment	in PBMCcompartment		
1	41	Plasma	0	8	5	12%
2	39	PBMC	11	2	3	8%
3	48	PBMC	5	4	48	100%
4	39	Plasma	6	13	5	13%
5	30	PBMC	4	3	5	17%
6	45	PBMC	58	6	5	11%
7	43	PBMC or Plasma	1	1	43	100%
8	50	PBMC	16	2	7	14%

PBMC = peripheral blood mononuclear cells.

### Characterization of Coreceptor Tropism of Transmitted/founder Viruses

HIV-coreceptor usage determination using SVM_Geno2pheno10%_ showed that all donors were predominantly infected with CCR5-tropic strains (“R5-strains”). However, there was a minority of CXCR4/dual-mixed (“X4/DM” strains) viruses among DNA and/or RNA sequences in 4/8 donors; these strains represented 2.1% (donor#3), 2.6% (#2), 16.7% (#5) and 22.0% (#8) of the viral quasispecies.

Again, the viral quasispecies identified in the eight recipients were highly homogeneous and displayed exclusive CCR5-tropism in 6/8 cases and exclusive X4/DM-tropism in 2 cases (recipients#5 and 8). The two patients harboring X4/DM viruses were women infected through heterosexual contact each with a donor harboring a minority of X4/DM-tropic strains (6.7% of DNA and 26.7% of RNA sequences for donor#5; 16.0% of DNA and 28.0% of RNA sequences for donor#8). The proportion of X4/DM quasispecies in donors was higher in cases of X4/DM than R5 HIV transmission (16.7–22.0% versus 0–2.6%).

## Discussion

We report an analysis of eight transmission pairs, each including a sexually infected patient enrolled into the French ANRS PRIMO cohort at the time of primary HIV-1 infection and his/her donor. This provided a unique opportunity to compare epidemiologically linked virus populations in donors and recipients very close to the time of virus transmission, contrary to many previous studies which only characterized the transmitted viral populations in recipients without available virological data in donors.

Our study was based on the novel SGA-based approach involving more precise methods than those used in many previous studies for estimating the multiplicity of HIV-1 infection in recently infected individuals [3], [5], [7], [8], [10]–[12], [36], [37]. We found that the viral populations, as assessed from both DNA and RNA sequences, were highly homogeneous in each of the eight sexually PHI patients. Our findings are consistent with recent studies, based on similar methods, which suggest that there is a severe genetic bottleneck associated with sexual transmission of HIV-1 [13], [14], [16], [17]. The observed highly homogeneous viral population in PBMC is consistent with a single variant being transmitted, which then massively fuels the cellular reservoir, rather than multiple variants being transmitted rapidly followed by virus population homogenization in the recipient [37].

We herein show that the donor variant most closely related to the strain establishing infection was infrequently found within the quasispecies present in donor blood. This result suggests that the recipient’s infection is not due to the preferential transmission of a strain overrepresented among circulating quasispecies in donor. There are several possible explanations for this finding. First, the viral transmission could result from a stochastic process of a donor’s variant whatever its frequency among blood quasispecies. Second, the severe genetic bottleneck which occurs during sexual transmission, involves properties of the “mucosal barrier” and/or selection of blood variants with properties favoring transmission, as recently suggested [16]. In both hypotheses, we cannot exclude that compartmentalization between blood and genital viral subpopulations may contribute to the selection of the transmitted/founder strain: at the time of transmission, the predominant strains may be different in the blood and genital compartments. Boeras *et al* recently analyzed viral envelope sequences in the blood and genital fluids of eight transmission pairs and found that the viruses establishing infection were in most cases more closely related to blood-derived variants than to the variants prevalent in the genital compartment [38]. However, a recent study in the SIV macaque model suggested that SIV sequences are intermixed between the blood and the semen at the time of peak virus replication, but that SIV replication evolves to compartmentalization in the male genital tract after peak viremia resolves [39]. Similarly, Redd *et al* working with Ugandan HIV-1-discordant couples showed preferential transmission of ancestral as opposed to contemporary strains circulating in the donor [40]. The authors suggested that transmitted strains may be sequestered in a long-lived reservoir during the early stage of infection, such as latently infected cells of the genital tract and persist at a low level in blood and are potentially preferentially selected for subsequent transmission.

In 2010, a retrospective comparison of multivariant HIV-1 transmission among patient cohorts using new SGA-based determinations concluded that the multiplicity infection is higher in MSM than in heterosexuals (38% versus 19%) [17], consistent with previous studies using less precise methods [36]. We report an homogeneous viral population in each of eight sexually infected patients enrolled near the time of PHI, including four male-to-male, three male-to-female and one female-to-male transmissions. The small number of MSM donors included in our study and the fact than one of them (donor#7) harbored a homogeneous viral population may explain the absence of differences between MSM and heterosexuals. However, our findings are in line with a much larger study of clonal *env* sequences obtained from 145 patients at the time of PHI, which suggested that homosexual (versus heterosexual) transmission mode did not predict transmission of more heterogeneous founder virus populations [41].

In addition, we did not evidence any case of multiple infection in patients with STI, although 3/8 recipients reported STI in the 6 months prior to PHI diagnosis and 2/8 donors tested positive for syphilis at the time of blood collection. These findings are in line with the Rieder’s study, which did not find elevated complexity of transmitted viruses in patients infected through sexual intercourse and presenting with a concomitant STI during PHI [41]. In contrast to these results, Haaland *et al* reported that multiple variant transmission was associated with the presence of genital inflammation or ulceration or with self-reported lower abdominal pain in 42 recently infected heterosexuals [16]; however, the authors did not find any association between multiple variant transmission and vaginal/urethral discharge, cystitis or the presence of genital inflammation or genital ulceration when analyzed as independent risk factors. Sagar *et al* previously suggested that the presence of genital tract infections was associated with the acquisition of multiple variants in Kenyan female sex workers [42]. However, samples from the sexual partners of these patients were not available in this study, so it is not clear whether some of these multiple infections were or were not due to successive infections rather than to concomitant transmission of multiple variants. Further studies including samples from both donors and recipients are needed to characterize the type of genital disease susceptible to increase the risk of multiple variant transmission.

The eight donors in our study were predominantly infected by R5-tropic strains, but two recipients were infected with a homogeneous X4/DM viral population, isolated in both DNA and RNA samples. Interestingly, the proportions of X4/DM viruses in the viral quasispecies in their respective donors were significantly higher than those in the other donors. There are at least two possible explanations for these findings. First, our cases of X4-tropic viral transmission may have been driven by a disproportionately higher proportion of X4 strains in the donor’s genital fluids than in the blood compartment. However, this is not consistent with previous reports, which indicate that the frequency of X4/DM quasispecies in both male [43] and female [44] genital tracts are lower than in blood plasma. Second, the transmission of X4/DM variants could result from a stochastic process. This would be inconsistent with the conclusions of numerous studies which have attempted to correlate the predominance of CCR5 strains during the acute phase of infection with a biological bottleneck inherent to the genital mucosa [45], [46]. However, no conclusive evidence has been provided to indicate that X4 viruses are less transmissible [47] and a recent study concluded that R5 and X4-infections may result from a stochastic process [48]. This conclusion is coherent with our results, which suggest that the transmission of X4 strains is associated with a threshold population of X4 quasispecies in donor plasma and PBMC samples.

That a single virus, derived from an infrequent variant of the donor quasispecies, establishes infection in patients confirms that a severe genetic bottleneck occurs during subtype B HIV-1 heterosexual and homosexual transmission. Additional studies are required to fully understand the traits that confer the capacity to transmit and establish infection, and determine the role of concomitant STIs in mitigating the genetic bottleneck in mucosal transmission. Such studies will be critical for guiding interventions aimed at preventing HIV-1 sexual transmission.
